# Failure Analysis and Optimized Simulation Design of Silicon Micromechanical Resonant Accelerometers

**DOI:** 10.3390/s25154583

**Published:** 2025-07-24

**Authors:** Jingchen Wang, Heng Liu, Zhi Li

**Affiliations:** School of Electronics and Information Engineering, Nanjing University of Information Science and Technology, Nanjing 210044, China; 002357@nuist.edu.cn (H.L.); 202312490153@nuist.edu.cn (Z.L.)

**Keywords:** accelerometer, failure analysis, finite element simulation, stress isolation

## Abstract

To develop solutions to the frequency instability and failure of silicon micromechanical resonant accelerometers, the state characteristics of micromechanical resonant accelerometers are investigated under temperature and vibration stresses. Through theoretical analysis and finite element simulation, the following is found: the Young’s modulus of silicon varies with temperature, causing a resonance frequency shift of −1.364 Hz/°C; the residual stress of temperature change affects the resonance frequency shift of the microstructure, causing it to be 5.43 Hz/MPa (tensile stress) and −5.25 Hz/MPa (compressive stress); thermal expansion triggers the failure of the bonding wire, and, in the range of 10 °C to 150 °C, the peak stress of the electrode/lead bond area increases from 83.2/85.6 MPa to 1.08/1.28 GPa. The failure mode under vibration stress is resonance structure fracture and interlayer peeling. An isolation frame design is proposed for the sensitive part of the microstructure, which reduces the frequency effects by 34% (tensile stress) and 15% (compressive stress) under temperature-variable residual stresses and the maximum value of the structural root mean square stresses by 69.7% (X-direction), 63.6% (Y-direction), and 71.3% (Z-direction) under vibrational stresses.

## 1. Introduction

Micromechanical accelerometers are widely used in both civil and military applications, including inertial navigation, consumer electronics, and seismic wave detection. This is due to their advantages of small size, low power consumption, and suitability for mass production [1]. Micromechanical resonant accelerometers are sensitive to acceleration through the frequency change of the resonant beam, and their output frequency signal exhibits better anti-interference capabilities than that of micromechanical capacitive force-balanced accelerometers, which represents an important development direction for inertial devices. Micromechanical resonant accelerometers primarily utilize quartz and silicon-based materials, with silicon being more mature in its manufacturing process. They can be integrated with the interface circuit on the same substrate. In contrast, the interface circuit of quartz micromechanical resonant accelerometers is independent, corresponding to the relatively large volume of the accelerometer system. Currently, more silicon micromechanical resonant accelerometers are being developed, as the interface circuit’s signal-to-noise ratio is low [2]. The structural layer of silicon micromechanical resonant accelerometers is generally made of silicon-based material, and it has been shown that accelerometers are more sensitive to thermal and vibrational stresses than other stresses [3]. Temperature changes lead to changes in the Young’s modulus and coefficient of thermal expansion of silicon-based material, both of which affect the stiffness of the beam, resulting in temperature-dependent deviations of the resonant frequency. In the long-term operation of silicon micromechanical resonant accelerometers, the temperature sensitivity of silicon not only reduces the measurement accuracy of the accelerometer but also induces structural failures, such as resonator failure and fracture. Under extreme operating conditions, the temperature nonlinearity of the material properties also has a coupling effect on the failure mechanism [4]. Under vibration stress, beams or thin-film structures may overbend, leading to adhesion, fracture, or delamination on the device surface. The reciprocating motion of the structure caused by long-term environmental vibration may even lead to fatigue failure [5].

Currently, the failure study of silicon micromechanical accelerometers focuses primarily on capacitive force-balanced accelerometers, whose failure judgment is based on the signal stability of the amplitude of the capacitance change and the integrity of the mechanical structure [6]. A resonant accelerometer measures acceleration by detecting the change in the vibration frequency of the resonant beam, and its failure modes (e.g., resonant frequency drift and vibration mode instability) are fundamentally different from those of the capacitive type: the former requires characterizing acceleration by detecting the frequency change of capacitance signals in the resonant state, while the latter relies directly on the static amplitude of the change in the capacitance pole–plate spacing [7]. Although a silicon micro-resonant accelerometer also measures frequency through capacitance detection, the stability of its closed-loop resonant system not only depends on the capacitance signal characteristics but also closely relates to the dynamic characteristics of the resonant beam (e.g., quality factor and modal frequency). There is a lack of systematic research in the existing literature on the key issues of a resonant device’s frequency drift mechanism and failure [8].

Currently, domestic and international research on optimization methods for silicon micro-resonant accelerometers primarily focuses on two major technologies: passive technology and active technology. The passive technique is mainly aimed at optimizing the design of the resonator. Active techniques typically require additional circuits, temperature sensors, or resonator components, and the control stability is weak [9]. In contrast, passive techniques can significantly reduce the inherent dependence of the resonant frequency on sensitive stresses, eliminating the need for external circuits and offering the advantages of low power consumption and high reliability [10]. For temperature stresses, the approaches can be categorized into two directions: reducing the frequency temperature coefficient of the resonator [11] and reducing the structural thermal stresses [12]. Synergistically minimizing the effects of thermal stress and vibration stress has become a key challenge in addressing the stability of silicon microaccelerometers in complex environments.

This paper conducts a characteristic analysis and failure study of silicon micro-resonant accelerometers under thermal stress and vibration stress. Frequency quantitative models are established under multiple factors, and finite element analysis is used to identify failure modes. Based on the identified weak links, the microstructure is optimized and improved.

## 2. Failure Mode and Mechanism Study Under Temperature Stress

**Figure 1:**
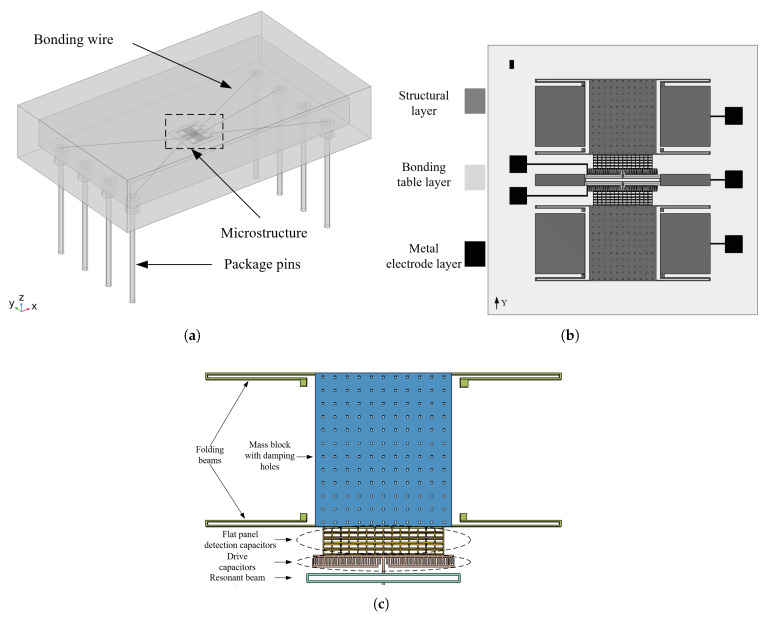
(**a**) Schematic diagram of the overall structure of the accelerometer. (**b**) Microstructure diagram. (**c**) Structural layer diagram.

As shown in Figure 1, a silicon micromechanical resonant accelerometer as a whole consists of a table core, a lead, and a housing package, and the table core includes a silicon structure layer, a gold electrode layer, and a bonding table layer. The dimensional parameters of the packaging shell are shown in Table 1. The silicon micromechanical resonant accelerometer is sensitive in the Y-direction. The accelerometer core structure adopts a single-sided electrostatic drive and a single-sided flat capacitance detection method. When the acceleration is 0, the resonant beam is in a resonant state after power-on; when the acceleration is not 0, the inertial force displaces the sensitive mass block and its attached flat plate, the detection of the flat plate and the flat plate on the resonant beam spacing changes, and the electrostatic negative stiffness changes. The result leads to a change in the resonant beam resonance frequency, which is the principle behind the resonance microaccelerometer. The encapsulated housing of the accelerometer is made of Kovar alloy. Kovar alloy is an iron–nickel–cobalt alloy with a low thermal expansion coefficient, which is essentially unaffected by temperature stresses and is therefore not considered to produce failures [13].

**Figure 2:**
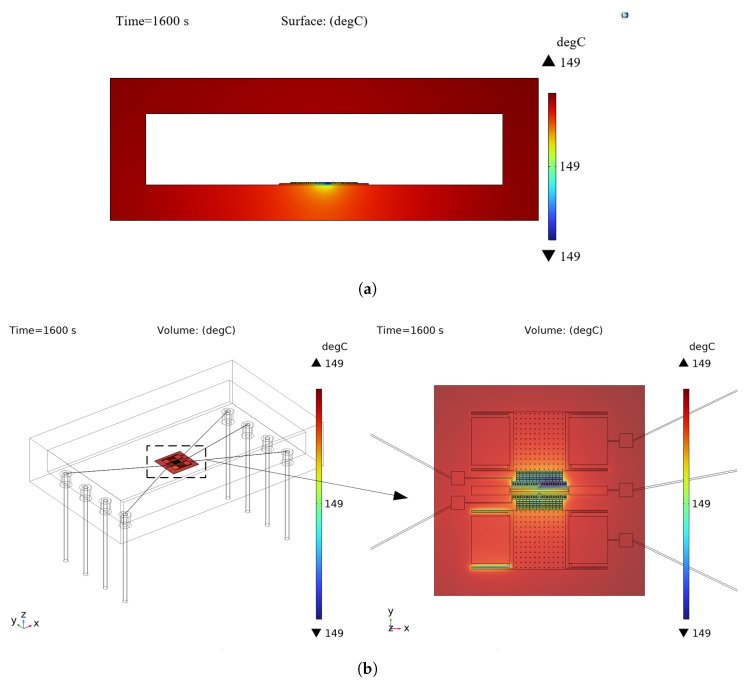
Under conditions of an initial temperature of 10 degrees Celsius and an ambient temperature of 150 degrees Celsius, the temperature of the accelerometer at 1600 s. (**a**) Overall temperature profile of the accelerometer. (**b**) Microstructure temperature distribution map.

**Figure 3:**
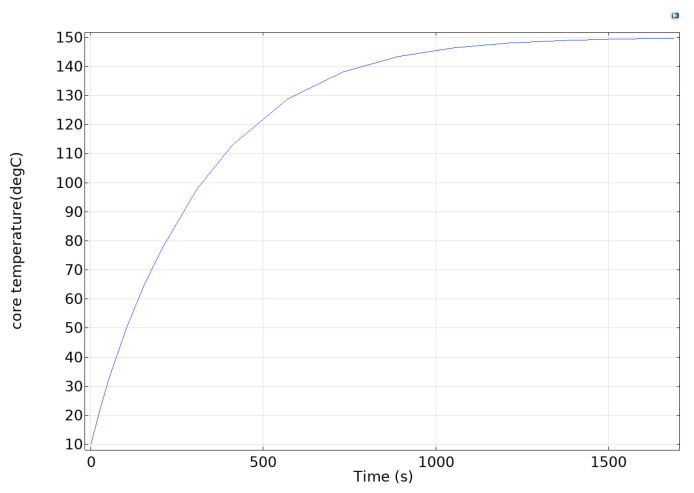
Microstructure heating change curve.

It is crucial to simulate the internal temperature variation of the accelerometer before failure analysis, and dynamic heat transfer data can not only pinpoint the internal thermal stress layout but can also provide excitation input for subsequent finite element simulations. A 3D thermodynamic model of the accelerometer is established using COMSOL Multiphysics 6.3, and its temperature field evolution in a high-temperature environment (150 °C) is simulated through transient heat transfer analysis. Thermal convection boundary conditions are used to characterize the external air in the simulation, while the heat conduction between materials and the thermal radiation effect inside the cavity are considered. The results of the transient study show that the accelerometer core exhibits a prominent non-uniform temperature field distribution characteristic under thermal loading. From the heat distribution (shown in Figure 2) and the temperature change curve of the structural layer (shown in Figure 3), it can be seen that the transient thermal response rate of the microstructure exhibits a nonlinear decay trend with the continuous action of the thermal conditions. Its temperature gradient tends to maintain a steady state with the extension of the thermal loading time, providing a load input for the subsequent failure mode analysis.

### 2.1. Changes in Young’s Modulus Can Cause Performance Failures

Both the power-up and temperature changes in the environment affect the temperature of the silicon material, and the Young’s modulus of the silicon material is affected by the temperature change, resulting in a temperature drift of the accelerometer performance [14]. According to existing research [15], the Young’s modulus *E* of silicon corresponds to the temperature *T* as follows:

(1) E=−0.0096T+130.25

where the unit of *E* is GPa. The resonant beam stiffness is

(2) $$k=\frac{256E\times w^{3}\times h}{15L^{3}}$$

where *L*, *w*, and *h* are the length, width, and height of the resonant beam.

Based on the principle of electrostatic negative stiffness, the resonance frequency model is established as follows:

(3) $$f_{e}=\frac{1}{2\pi }\sqrt{\frac{k-k_{e}}{m}}=\frac{1}{2\pi }\sqrt{\frac{\frac{256\times \left(-0.0096T+130.25\right)\times w^{3}\times h}{15L^{3}}-\frac{\varepsilon AV_{S}^{2}}{{\left(g_{0}-x\right)}^{3}{\left(1-\frac{-\Delta x}{g_{0}-x}\right)}^{3}}}{m}}$$

where $k_{e}$ is the electrostatic negative stiffness, ε is the vacuum dielectric constant inside the packaging shell, *A* is the detection area of the detection plate capacitance, $V_{s}$ is the DC detection voltage connected to the detection plate capacitance system, $g_{0}$ is the initial Y-direction spacing between a single detection capacitance and the resonator, and *m* is the equivalent mass of the vibration modes of the resonant beam. The specific values are shown in Table 2.

**Figure 4:**
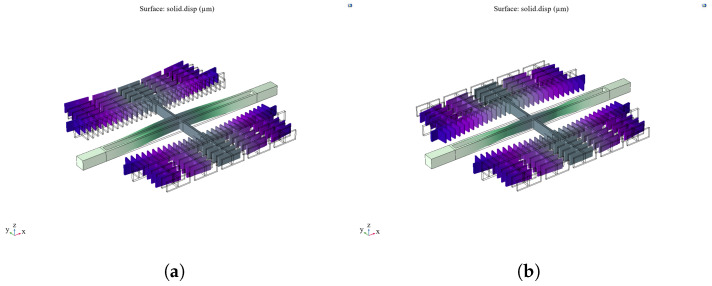
Vibration modes of accelerometers. (**a**) First-order in-plane isotropic modes. (**b**) First-order in-plane reversed modes.

**Figure 5:**
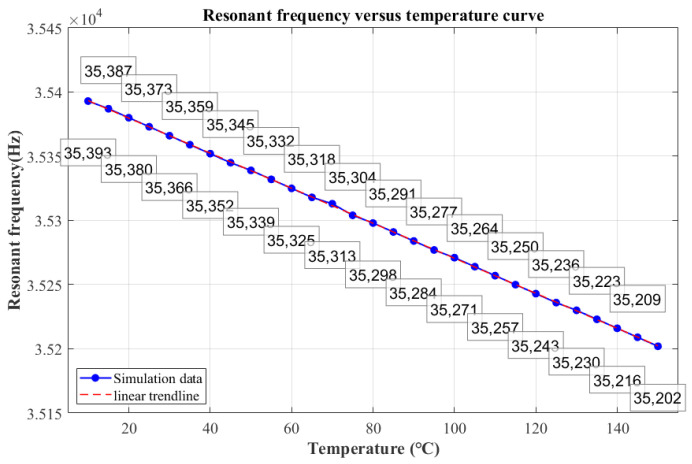
Resonance frequency variation curve with temperature, considering changes in Young’s modulus.

According to existing studies [16], it is calculated that Young’s modulus decreases with an increasing temperature, and the resonant frequency also decreases. The above text innovatively derives the quantitative mechanism by which changes in Young’s modulus affect the resonant frequency, and the theoretically calculated value of frequency drift is −1.309 Hz/°C. Finite element analysis is performed on the resonant structure to obtain its vibration modes, as shown in Figure 4. The Young’s modulus of silicon material varies with temperature, and the resonance frequency of the accelerometer at different temperatures is obtained. The simulation data under the influence of Young’s modulus is recorded in Figure 5, and the frequency drift is −1.364 Hz/°C. Combining the simulation with the temperature characterization test, it can be seen that the frequency change caused by the temperature change is less than 200 Hz within the range of 10 °C to 150 °C. Measurement failure is triggered when the cumulative frequency deviation exceeds 200 Hz.

### 2.2. Residual Stress-Induced Performance Failures

**Figure 6:**
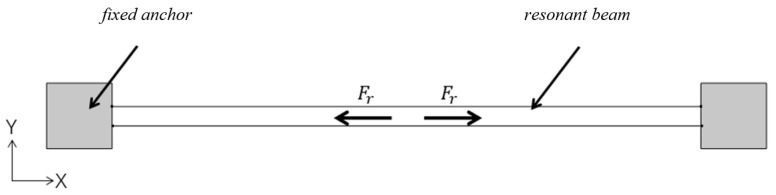
Schematic representation of the residual stresses in the accelerometer without electrostatic drive.

Temperature-dependent residual stress effects are introduced into the silicon-based material structure during the manufacturing and processing flow of accelerometers [17]. Compared to folded beam structures, resonant beams are more sensitive to such residual stresses due to their unique dynamics. Considering small deformations and neglecting shear deformations and transverse inertia, the Euler–Bernoulli beam model is used. The main manifestation of residual stresses in silicon structures is normal stresses. Assuming that the residual stresses in the beam are tensile stresses with a positive direction (the direction of compressive stresses is negative), when there is no electrostatic drive, as in Figure 6, the magnitude of the residual stresses in the beam is

(4) $$F_{r}=\sigma_{r}\left(1-\mu \right)wh$$

where $\sigma_{r}$ is the residual stress. μ is the Poisson ratio of silicon, with a value of 0.28.

**Figure 7:**
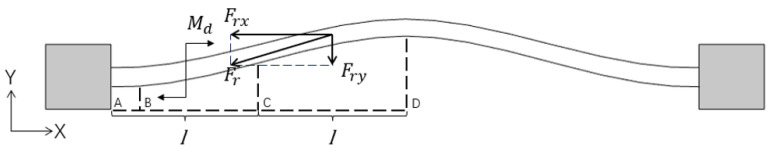
Schematic illustration of accelerometer residual stresses during electrostatic driving.

When the driving force is applied, the beam will bend, with point B as the center of bending, and the resonant beam will undergo constant-amplitude vibration. Since the structure is left–right symmetric, only the left half is analyzed. The shape of the beam in the driving mode is shown in Figure 7. Under the action of the bending moment, the beam deforms, resulting in a residual stress with a component in the Y-direction. Since the deformation of the beam is minimal, in the static analysis of the beam in the AD section, the moment generated by the residual stress component in the BD section of the beam is equal to the external moment, which can be obtained as follows:

(5) $$M_{d}=\int_{0}^{2l}F_{r}\sin\theta dx$$

where *l* is a quarter of the length of the resonant beam.

Considering that the angle of rotation is slight, it can be determined as

(6) $$\sin\theta \approx \theta =\frac{M_{d}}{2lF_{r}}=\frac{M_{d}}{2l\sigma_{r}\left(1-\mu \right)wh}$$

Therefore, the bending stiffness due to residual stress is

(7) $$k_{re}=\frac{M_{d}}{\theta }=2l\sigma_{r}\left(1-\mu \right)wh$$

After considering the residual stresses, the total stiffness of the resonant beam is expressed as

(8) $$k^{\prime }=k+k_{re}=\frac{256E\times w^{3}\times h}{15L^{3}}+2l\sigma_{r}\left(1-\mu \right)wh$$

The resonant frequency is

(9) $$f_{e}=\frac{1}{2\pi }\sqrt{\frac{k^{\prime }-k_{e}}{m}}=\frac{1}{2\pi }\sqrt{\frac{\frac{256E\times w^{3}\times h}{15L^{3}}+2l\sigma_{r}\left(1-\mu \right)wh-\frac{\varepsilon AV_{S}^{2}}{{\left(g_{0}-x\right)}^{3}{\left(1-\frac{-\Delta x}{g_{0}-x}\right)}^{3}}}{m}}$$

**Figure 8:**
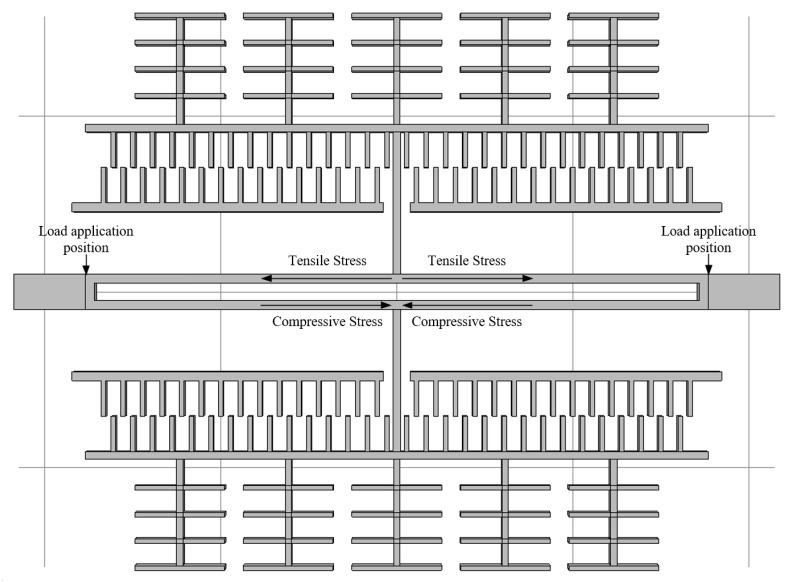
Schematic diagram of the physical field setup.

**Figure 9:**
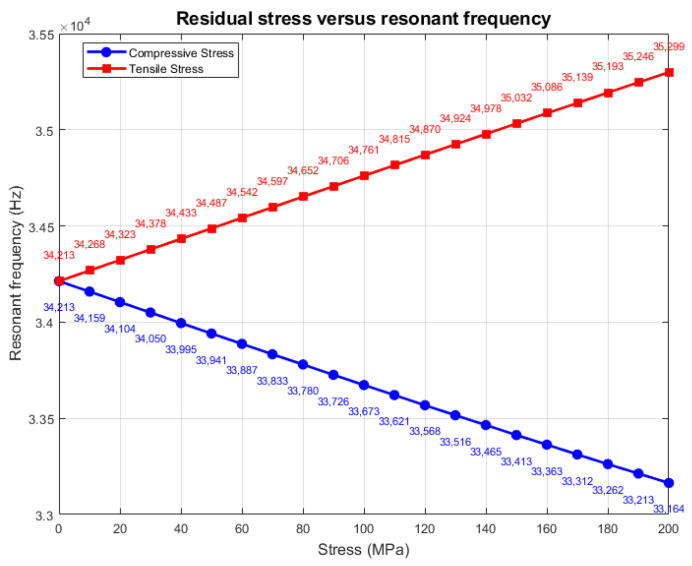
Analysis of resonant frequencies under the influence of residual stresses.

The above derivation (Formulas (4)–(9)) quantifies the mechanism by which residual stress affects the resonance frequency. From the formulas, it can be seen that the theoretical resonant frequency change under tensile stress is 5.37 Hz/MPa, and the theoretical resonant frequency change under compressive stress is −5.23 Hz/MPa. A boundary load is added to the contact surface between the resonant beam and the fixed anchor point, and the load directions of the compressive and tensile stresses are shown in Figure 8. The load is set to be from −200 MPa to 200 MPa, where − denotes that the residual stress is compressive, and + denotes that the residual stress is tensile. Stress and + indicate that the residual stress is tensile. The specific results of the simulation are shown in Figure 9, and the analysis reveals that the resonance frequency changes to 5.43 Hz/MPa under tensile stress and −5.25 Hz/MPa under compressive stress, which is in close agreement with the theoretical calculation.

### 2.3. Structural Failure Due to Thermal Expansion Effects

Due to the mismatch between the thermal expansion coefficients of different materials, these materials expand to varying degrees under thermal stress, causing the structure to deform, delaminate, and potentially fail. In the accelerometer core, monocrystalline silicon is used for the structural layer, gold is used for the electrode layer, and Pyrex 7740 glass is used for the backing material. When the multi-layer structure is subjected to temperature stress, the difference between the thermal expansion coefficients of the materials leads to stress concentration on the contact surfaces and peeling at the bonding interface. Meanwhile, after the encapsulation of the core, the gold wire leads in the resin deform and delaminate to different degrees due to the thermal expansion effect. Table 3 shows the coefficients of thermal expansion that lead to the deformation of materials due to thermal expansion, which can be expressed as

(10) εT=α×ΔT

where εT is the thermal strain, α is the coefficient of thermal expansion, and ΔT is the temperature change.

**Figure 10:**
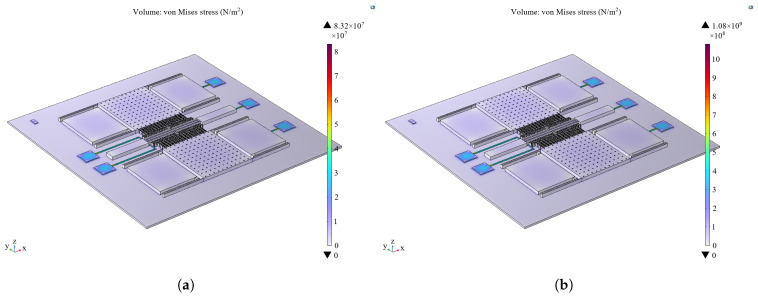
(**a**) Finite element analysis of thermal expansion of microstructures at 10 °C. (**b**) Finite element analysis of thermal expansion of microstructures at 150 °C.

The thermal expansion effect is incorporated into the multiphysics field. The bottom surface of the bonding table layer is set as a fixed constraint, and the core temperature is adjusted according to the temperature gradient to analyze the stress distribution resulting from thermal expansion. The thermal expansion effect mainly occurs in the metal electrode region (shown in Figure 10), and the maximum value varies from 83.2 MPa to 1.08 GPa in the range of 10–150 °C. In contrast, the thermal expansion effect of the bonding table layer and the structural layer is small and almost negligible.

**Figure 11:**
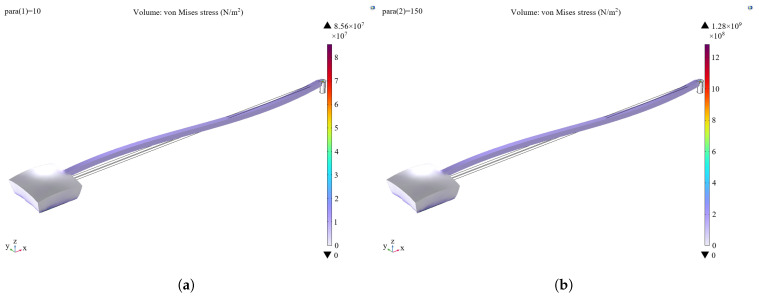
(**a**) Finite element analysis of thermal expansion of gold wire bonding at 10 °C. (**b**) Finite element analysis of thermal expansion of gold wire bonding at 150 °C.

The structure temperature is set according to the temperature gradient to analyze the stress distribution of the leads under thermal expansion. As can be seen in Figure 11, the stresses are mainly distributed at the metal electrode, the metal electrode bonding, and the overall lead, and the maximum value varies from 85.6 MPa to 1.28 GPa in the range of 10–150 °C. The failure due to thermal expansion is characterized by thermal deformation and damage to the metal electrode and metal lead part.

## 3. Failure Mode and Mechanism Study Under Vibration Stress

Common vibration situations are categorized into sinusoidal and random vibration. A single frequency characterizes sinusoidal vibration and cannot accurately represent multi-frequency vibration situations in real environments. In contrast, random vibration testing is more closely aligned with the vibration experienced in real-world environments. It can excite multiple modes simultaneously, which may be more effective in exposing the failure modes of sensors under multi-frequency stresses. Fatigue accumulation damage occurs when accelerometers are subjected to vibration stress for an extended period, which is calculated using an S−N curve based on Basquin’s law [20]:

(11) $$N=C\times {\left(\Delta S\right)}^{m}$$

where *N* denotes the number of cycle lifetimes, *S* denotes the stress variation, and *C* and *m* are the fatigue strength coefficient and fatigue strength index of the material. The Basquin fatigue model parameters are shown in Table 4 [21,22,23,24].

A deterministic time-domain function cannot describe random vibrations; their statistical properties are usually characterized in the frequency domain using the power spectral density function, with the power spectral density parameters listed in Table 5.

Root mean square (RMS) stress is a crucial statistical parameter that characterizes the response of a structure to random vibration, quantifies the time-averaged intensity of vibration energy, and reflects the long-term fatigue damage trend of the structure.

(12) $$\sigma_{\mathrm{rms}}=\sqrt{\int_{f_{1}}^{f_{2}}PSD_{\sigma }\left(f\right)df}$$

where $f_{1}$ and $f_{2}$ are the lower and upper limits of the frequency range, and $PSD_{\sigma }\left(f\right)$ is the power spectral density of the stress. Vibratory loading as a zero-mean symmetric cycle can be considered as

(13) $$S=\sigma_{rms}$$

From the formulas, it can be seen that the point in the structure with the smallest number of cycle lifetimes is subjected to the most considerable RMS stress and is the weakest link in the structure.

### 3.1. Structural Fatigue and Delamination Due to Long-Term Vibration

**Figure 12:**
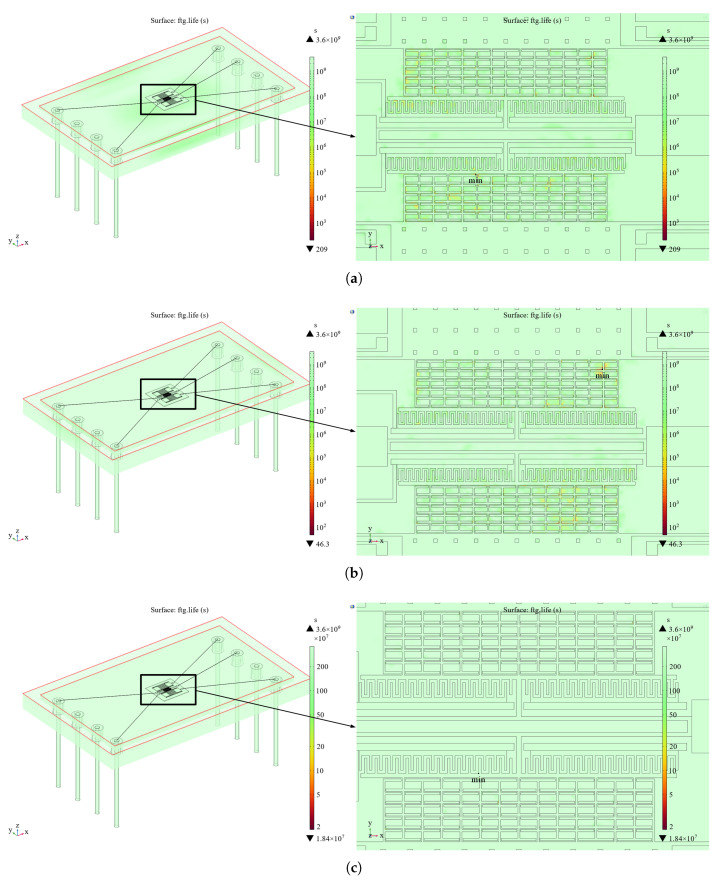
Fatigue analysis of the random vibration of the accelerometer as a whole. (**a**) In the X-direction. (**b**) In the Y-direction. (**c**) In the Z-direction.

According to the model in Figure 1, random vibration is applied to the accelerometer as a whole in the X-, Y-, and Z-directions in COMSOL Multiphysics software. The resonant frequency of the resonant accelerometer is significantly larger than 2 kHz, which satisfies the assumption of a rigid connection and allows for the neglect of full-domain coupling in the dynamic response of the microstructure. Subsequently, vibration analysis is carried out on the microstructure. As shown in Figure 12, the minimum fatigue life points in the three directions of vibration are all at the bonding of the resonant structure, in which the RMS stress at the minimum fatigue life point is 54.90 MPa when vibrating in the X-direction, 106.79 MPa when vibrating in the Y-direction, and 15.45 MPa when vibrating in the Z-direction, so the failure mode is a long-term vibration of the structural layer and the structural layer with a rigid connection [25]. Therefore, the failure mode is structural failure resulting from the detachment of the structural layer and bonding layer due to long-term vibration.

### 3.2. Microstructural Beam Fracture

**Figure 13:**
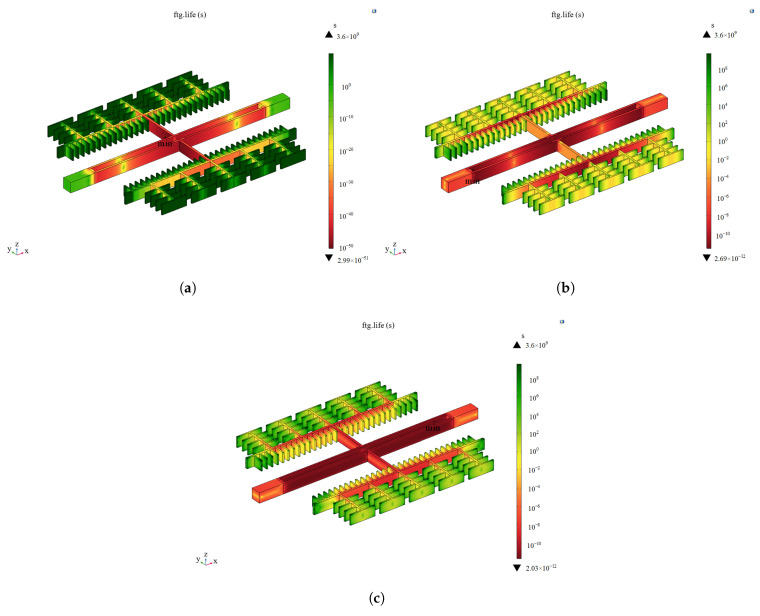
Failure cycle analysis of resonant structures under random vibration. (**a**) In the X-direction. (**b**) In the Y-direction. (**c**) In the Z-direction.

The previous section simplified the failure of the microstructure by treating it as a rigid connector, and it was found that the resonating structure exhibited a stress concentration phenomenon. Therefore, the resonating structure was further analyzed. A random vibration load was directly applied to the resonant structure, and the results are shown in Figure 13. The minimum fatigue life points in the three directions of vibration are all in the resonant beam, in which the maximum value of the structural RMS stress is 279.7 MPa in the X-direction, 176.5 MPa in the Y-direction, and 221.5 MPa in the Z-direction. It can be seen that the weak link is the resonant beam, and the failure mode is the fracture of the resonant beam.

## 4. Microstructure Optimization Design

**Figure 14:**
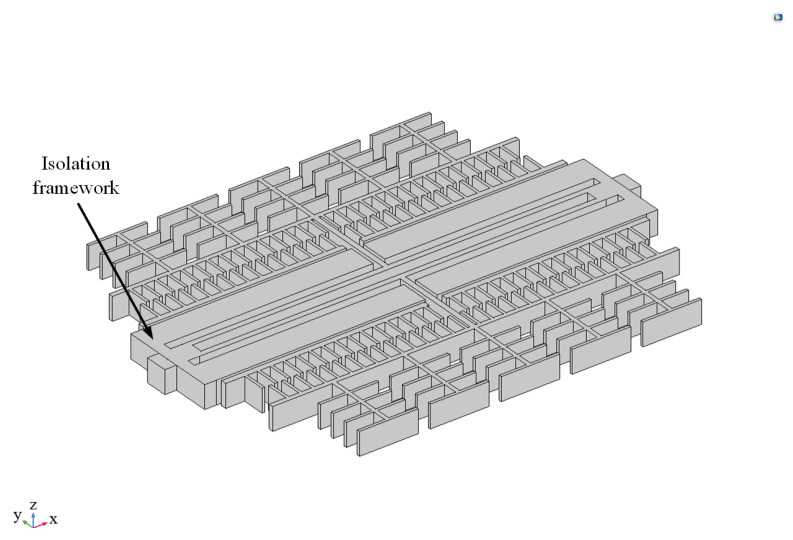
Microstructure optimization design.

A microstructure optimization design is proposed to address the residual stresses that affect the resonant frequency of accelerometers under temperature stresses and the fracture of resonant beams under vibration stresses. According to the principle of the spring–mass system, a low-stiffness structure absorbs the thermal expansion and vibration energy, reducing the stress transferred to the resonant beam. A set of stress-isolating frames is added outside the maximum displacement of the resonant beam, which is modeled as shown in Figure 14. The dimensions are listed in Table 6.

### 4.1. Structural Feasibility Analysis

**Figure 15:**
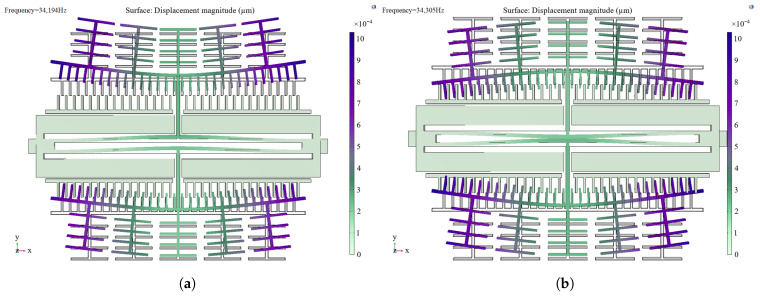
(**a**) First-order in-plane isotropic modes of the modified resonant structure. (**b**) First-order in-plane reversed modes of the modified resonant structure.

Firstly, modal analysis of the resonator structure is carried out, and its first-order in-plane isotropic and first-order in-plane inverse modes are 34,194 Hz and 34,305 Hz, respectively, after modal simulation, as shown in Figure 15. The analysis shows that the isolation frame design does not change the original resonant frequency or resonant mode of the microstructure.

Secondly, according to the working principle of the resonant microaccelerometer, it can be inferred that the folded beam stiffness should be significantly less than the stiffness of the resonant beam. The simulation results of applying different accelerations in the sensitive direction are presented in Table 7.

Through the static analysis, the simulated stiffness of the resonant beam is found to satisfy the requirement that the resonant beam stiffness is 15 times greater than the folded beam stiffness, and the structural design follows the working principle of the microaccelerometer.

### 4.2. Comparative Analysis of Optimization Results Under Temperature Stress

**Figure 16:**
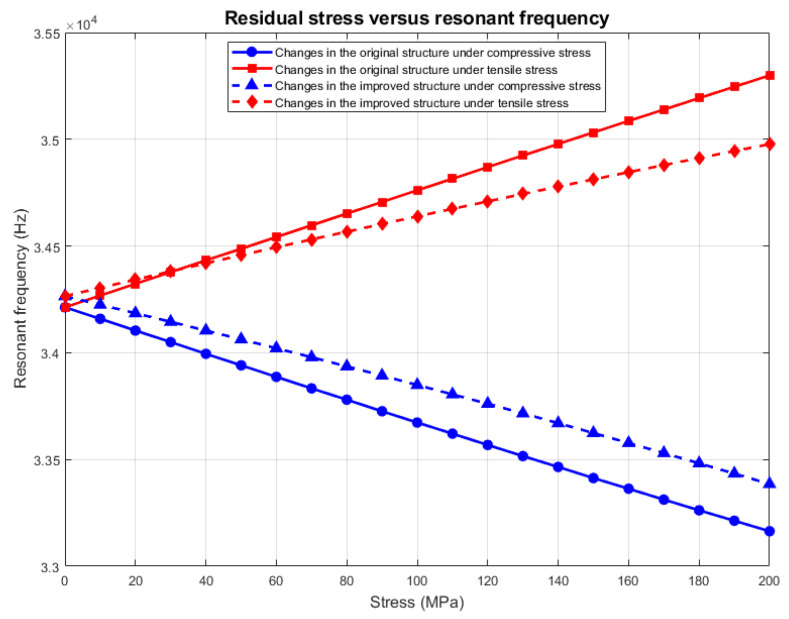
Effect of residual stress under temperature stress on modified resonant beams.

An analysis is conducted on the effect of residual stress on the improved resonant beam under temperature stress, and the results are compared with the analysis results of the original structure. The specific results are shown in Figure 16. When the change in the resonant frequency under tensile stress is 3.56 MPaHz/MPa, the frequency effect is reduced by 34%, and when the change in the resonant frequency under compressive stress is −4.4 Hz/MPa, the frequency effect is reduced by 15%; the results show that the improved structure minimizes the impact of temperature stress on the accelerometer microstructure failure.

### 4.3. Comparative Analysis of Optimization Results Under Vibration Stress

**Figure 17:**
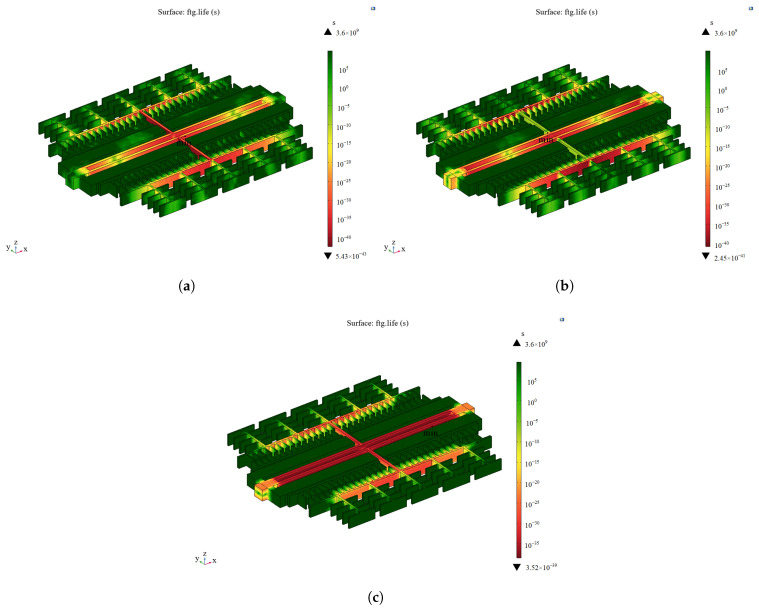
Failure cycle analysis of improved structures under random vibration. (**a**) In the X-direction. (**b**) In the Y-direction. (**c**) In the Z-direction.

The improved resonant structure is analyzed under vibration stresses, and the results are compared with the analysis results of the original structure. Figure 17 shows that the stress of the resonant structure is transferred from the resonant beam part to the isolation frame part. The minimum fatigue life point in the X-direction is on the resonant beam, and the maximum value of the structural RMS stress is 84.7 MPa, which is reduced by 69.7%. The minimum fatigue life point in the Y-direction is transferred from the resonant beam to the flat capacitor connecting beam, and the maximum value of the structural RMS stress is 64.3 MPa, which is reduced by 63.6%. In the Z-direction, the minimum fatigue life point is on the resonant beam, and the maximum value of structural RMS stress is 63.6 MPa, which is reduced by 71.3%. The results show that the improved structure minimizes the effect of vibration stress on the failure of the accelerometer microstructure.

## 5. Conclusions

Relevant failure analyses are carried out to address the frequency instability and failure of silicon micromechanical resonant accelerometers under temperature and vibration stresses. The frequency drift due to the Young’s modulus change with temperature is −1.364 Hz/°C; under the influence of residual stress, the resonance frequency change under tensile stress is 5.43 Hz/MPa, and the resonance frequency change under compressive stress is −5.25 Hz/MPa. Under the effect of thermal expansion, the peak stress at the electrode/lead bonding area in the range of 10 °C to 150 °C increases from 83.2/85.6 MPa to 1.08/1.28 GPa, leading to structural failure. Under vibration stress, the microstructure detaches from the bonding layer due to long-term vibration; the resonant structure within the microstructure is most affected by the stress and undergoes structural failure. An optimized design of microstructures based on isolation frames is proposed, which reduces the frequency impact by 34% under tensile stress due to temperature change and 15% under compressive stress. Under vibration stress, the stresses in the resonant beam in all three vibration directions are dispersed by the isolation frame, and the maximum values of the structural RMS stresses are reduced by 69.7%, 63.6%, and 71.3%. The results demonstrate that the improved structure mitigates the effects of temperature stress and vibration stress, enhancing the accelerometer’s stability in complex environments and providing a theoretical foundation for the design of highly reliable micromechanical accelerometers.

## Figures and Tables

**Table 1:** Dimension parameters of the packaging shell.

Length of packaging shell	Width of packaging shell	Height of packaging shell
20 mm	12 mm	4 mm
**Length of packaging pins**	**Pitch of packaging pins**	**Width of bonding wire**
8 mm	2.6 mm	25 μm

**Table 2:** Parameters for resonant frequency calculation.

ε	*m*	$g_{0}$	*A*
8.85 × 10^−12^ F/m	50.661 μg	2.5 μm	0.4518 μm^2^
$V_{s}$	L	w	h
5 V	700 μm	8 μm	40 μm

**Table 3:** Comparison of coefficients of thermal expansion [18,19].

Materials	Silicon	Pyrex7740 Glass	Gold
α (10^−6^·K^−1^)	2.6	2.8	17.1

**Table 4:** Basquin fatigue model parameters.

Material	Fatigue Strength Index	Fatigue Strength Factor (MPa)
Silicon	−0.14	1000
Pyrex7740 Glass	−0.11	60
Gold	−0.01	150
Kovar Alloy	−0.1	800

**Table 5:** Random vibration power spectral density parameters.

Condition	Parameter
Frequency range (Hz)	20–2000
Power spectral density ((m/s^2^)^2^/Hz)	3.2
Root mean square acceleration (m/s^2^)	14.2

**Table 6:** Isolation frame design parameters.

Condition	Size (μm)
Frame Structure Length	360
Frame Structure Width	40
Frame Structure Height	40
Frame-to-Resonant Beam Spacing	40

**Table 7:** Static analysis of a modified resonant beam.

Acceleration Magnitude (m/s^2^)	Displacement Size (μm)	Stiffness (N/m)
2	0.000050227	149.59
4	0.00010045	149.60
6	0.00015068	149.59
8	0.00020091	149.59
10	0.00025114	149.59

## Data Availability

The original contributions presented in this study are included in the article. Further inquiries can be directed to the corresponding author.
